# Multivariate Neural Representations of Value during Reward Anticipation and Consummation in the Human Orbitofrontal Cortex

**DOI:** 10.1038/srep29079

**Published:** 2016-07-05

**Authors:** Chao Yan, Li Su, Yi Wang, Ting Xu, Da-zhi Yin, Ming-xia Fan, Ci-ping Deng, Yang Hu, Zhao-xin Wang, Eric F. C. Cheung, Kelvin O. Lim, Raymond C. K. Chan

**Affiliations:** 1 Neuropsychology and Applied Cognitive Neuroscience Laboratory, Key Laboratory of Mental Health, Institute of Psychology, Chinese Academy of Sciences, Room 606, South Building, 16 Lincui Road, Beijing, Beijing, 100101 China; 2 Key Laboratory of Brain Functional Genomics, Ministry of Education, Shanghai Key Laboratory of Brain Functional Genomics (MOE & STCSM), East China Normal University, Room 213, Junxiu Building, 3663 North Zhongshan Road, Shanghai, 200062 China; 3 Department of Psychiatry, Cambridge Biomedical Campus, University of Cambridge, Cambridge, CB2 0SP UK; 4 Institute of Neuroscience, Shanghai Institutes for Biological Sciences, Chinese Academy of Sciences, 320 Yue Yang Road, Shanghai, 200031 China; 5 Shanghai Key Laboratory of MRI, East China Normal University, 3663 North Zhongshan Road, Shanghai, 200062 China; 6 Department of General Adult Psychiatry, Castle Peak Hospital, 15 Tsing Chung Koon Road, Tuen Mun, N.T. Hong Kong Special Administrative Region, China; 7 Department of Psychiatry, University of Minnesota, F282/2A West 2450 Riverside Avenue, Minneapolis, MN 55454 USA

## Abstract

The role of the orbitofrontal cortex (OFC) in value processing is a focus of research. Conventional imaging analysis, where smoothing and averaging are employed, may not be sufficiently sensitive in studying the OFC, which has heterogeneous anatomical structures and functions. In this study, we employed representational similarity analysis (RSA) to reveal the multi-voxel fMRI patterns in the OFC associated with value processing during the anticipatory and the consummatory phases. We found that multi-voxel activation patterns in the OFC encoded magnitude and partial valence information (win vs. loss) but not outcome (favourable vs. unfavourable) during reward consummation. Furthermore, the lateral OFC rather than the medial OFC encoded loss information. Also, we found that OFC encoded values in a similar way to the ventral striatum (VS) or the anterior insula (AI) during reward anticipation regardless of motivated response and to the medial prefrontal cortex (MPFC) and the VS in reward consummation. In contrast, univariate analysis did not show changes of activation in the OFC. These findings suggest an important role of the OFC in value processing during reward anticipation and consummation.

The orbitofrontal cortex (OFC) has received considerable attention for its role in value computation/representation and value/utilities comparison in decision-making tasks^1^,^2^,^3^,^4^ as well as in the absence of an overt decision-making^5^,^6^.

The OFC, occupying the ventral surface of the frontal part of the brain, is a relatively large and heterogeneous brain area in human (comprising Brodmann Areas (BA) 11, 12, 13, 14 and 47) and non-human primates (BA 10, 11, 12, 13, and 14)^7^,^8^. It receives inputs from various sensory modalities and has reciprocal connections with limbic, striatal and frontal areas. It has been suggested that the OFC is a key and multifunctional brain area in the reward network^7^,^9^. For example, complex or abstract reinforcers (i.e. money and social reward) are represented more anteriorly in the OFC than less complex reinforcers (i.e. food and erotic information)^7^,^10^,^11^,^12^. Moreover, the medial (mOFC) and the lateral (lOFC) orbitofrontal cortex differentially respond to rewarding and punishing events^13^,^14^. In an animal study, Rolls and colleagues^15^ have suggested that the different subpopulations of neurons in the OFC encode value across several modalities including taste and odour as well as visual cues of rewarding objects and faces. Furthermore, neurons of the rats’ OFC were found to encode reward value in a population of cells rather than by a single unit^16^,^17^,^18^. These findings suggest a possible heterogeneous functional/anatomical organisation and distributed neural representations of values within the OFC.

In conventional neuroimaging analysis, which mainly focuses on mapping the extent of the regional averaged changes in blood-oxygen-dependent level (BOLD) signal^19^, considerable smoothing and averaging are employed during pre-processing and statistical testing. This may reduce the sensitivity for detecting subtle changes in anatomically/functionally heterogeneous areas (i.e. OFC) during reward processing^20^,^21^,^22^. Multi-voxel pattern analysis (MVPA) may overcome this limitation by capturing fine-grained changes involved in the encoding of values^20^,^22^. Few studies have investigated how the human OFC encodes value (valence and magnitude) during reward anticipation and consummation using MVPA^23^,^24^,^25^ and findings had been mixed. Kahnt and his colleagues, using MVPA, had shown that distributed pattern in the mOFC represented reward value during both reward anticipation and consummation^23^. Tusche *et al*.^25^ reported that multivariate pattern in the ventral prefrontal cortex represented attractiveness of consumer products (cars), which could predict consumers’ future choices of purchasing. However, another studies suggested that valence rather than magnitude is represented in the central OFC (located between medial and lateral OFC, BA 11 and 13 26) during reward anticipation^24^. In different studies, different phases (anticipatory vs. consummatory) of reward processing and sub-regions (mOFC, vmPFC, and central OFC) were investigated, which may complicate the interpretation of OFC’s role in reward valuation. In the present study, we aimed to further examine whether the mOFC and the lOFC encode valence and magnitude information in the anticipatory and consummatory phases of reward processing.

Representational similarity analysis (RSA)^27^,^28^,^29^,^30^, which is one type of MVPA, was employed in this study to detect multivariate fMRI activation pattern in the OFC. RSA was developed based on the assumption that information encoded by the brain can be represented by the similarity between fMRI patterns associated with different experimental conditions. In order to capture value processing during reward anticipation and consummation, the monetary incentive delay task (MID) was employed^31^. In this task, participants were presented with a cue (circle or square) with a value information (i.e. win¥5.00, exchange rate at the time of experiment was approximately 1 US dollar = ¥6.2) and were required to wait for a short period (anticipatory phase) before responding to a target. Following the target, there was another waiting period which was defined as the anticipatory phase after making a response. Finally, feedback containing reward or punishment information was informed to participants based on their performance (consummatory phase, Fig. 1). There were two types of anticipatory phase in the MID task: before and after making a response. Based on the framework of anticipatory affect model by Knutson *et al*.^32^, the anticipatory phase before making a response is regarded as a more important period, which determines human’s reward anticipation and promotes future motivated behaviour. Based on this theoretical framework, we focused on value processing during this anticipatory phase. In addition, we also examined the anticipatory phase after making a response, because this phase is a purer anticipatory phase and less affected by the response preparation.

In order to test whether or not the OFC represents valence and magnitude information during the anticipatory and consummatory phase, we constructed models RDMs based on the affective property of cue stimuli reflecting hypothesis on different value information (valence and magnitude) during each phase. For example, for a model of valence, regardless of how much money was presented, patterns of win conditions are similar to each other but different from the patterns of loss conditions and vice versa. There were three types of model RDMs for each kind of value information: a simple model for overall value encoding; a simple model for specific value encoding (i.e. win and loss for the valence model, respectively) and a complex model for continuous value encoding. The simple model reflects that the value was encoded as “all or none” (either the same or different pattern between conditions) while complex models represent a graded difference between values (See Fig. 2). Then we performed the Spearman’s correlation between the brain RDMs in the OFC and the models reflecting different value representations to see how the OFC represented value information.

Next, we explored whether or not the fMRI patterns in the OFC were similar to the activation patterns in those regions that were traditionally associated with reward anticipation and consummation. Previous studies have suggested that the anticipation of primary rewards (i.e. pleasant taste, smell)^12^ and secondary rewards (i.e. monetary and social rewards)^11^,^33^ increases the activity in the ventral striatum (VS) and the anterior insular (AI). A meta-analysis conducted by Liu and colleagues including 65 studies and 1553 foci and has implicated a role of the VS and the AI in reward anticipation^4^. On the other hand, in a recent imaging meta-analysis comprising 35 imaging studies and 461 foci, Diekhof and colleagues^34^ found that the MPFC and the VS encode reward magnitude during the consummation of primary and secondary rewards. Another meta-analysis conducted by Knutson and Greer including 12 studies and 87 foci has also emphasized on the important role of the VS and MPFC in reward consummation^32^. Therefore, the VS and the AI during the reward anticipation as well as the medial prefrontal cortex (MPFC) and the VS during the reward consummation were chosen as traditional reference regions and compared with OFC in this study.

## Results

### Reaction time and subjective affective ratings

Participants responded to the target more quickly with an increase in monetary value, regardless of valence (win /loss), which was reflected by the significant main effect of magnitude (*F* (2, 44) = 18.87, *p* < 0.001) and non-significant valence × magnitude interaction (*p* = 0.24). For anticipatory experience, an interaction effect of valence × magnitude was observed in participants’ valence ratings (*F* (2, 21) = 20.657, *p* < 0.001). Participants reported more pleasantness and aversive feeling with increases in reward and punishment value, respectively. For consummatory experience, we observed a significant outcome × magnitude interaction in valence ratings (*F* (2, 44) = 56.968, *p* < 0.001), indicating that participants reported more pleasantness/aversive experience with increases in monetary value when participants received favourable/favourable outcomes. In terms of arousal, a main effect for magnitude was observed during both the anticipatory and the consummatory phase (anticipation: *F* (2, 21) = 20.272, *p* < 0.001; consummation: *F* (2, 21) = 20.526, *p* < 0.001), indicating that the participants were more excited with the increasing monetary value during both reward/punishment anticipation and consummation (see Supplementary Fig. 1 in Supplementary Materials).

### Conventional univariate fMRI analysis

We did not observe any significant main effects of valence, magnitude, outcome or any interaction effects on OFC activation during either the anticipatory or the consummatory phase. Consistent with the previous findings by Knutson *et al*.^32^, our whole brain analysis showed a significant main effect for magnitude on activation in the VS in the anticipatory phase before making a response, a main effect of magnitude on activation in the AI in the anticipatory phase after making a response and a significant main effect of outcome and valence on activation in the VS and the MPFC in the consummatory phase (*p*_FWE-corrected_ < 0.05 at the cluster level, Fig. 3; and see Supplementary Table 1, 2 and 3 in Supplementary Materials).

We then carried out small volume correction analysis within the mOFC (BA 11: regions defined by BA atlas implemented in SPM) and lOFC (BA 12 and 47) during each phase. We found that, during either anticipatory phase before or after making a response, there was no significant main effect for valence, main effect for magnitude or valence × magnitude interaction effect on the mOFC and the lOFC activities. During consummatory phase, we did not observe any significant main effect or interaction in the mOFC or the lOFC either. Also, similar findings were observed when we carried out the analysis based on the percentage of BOLD signal change in the OFC (See Fig. 2 in the supplementary material).

Therefore, consistent with the previous studies, the conventional univariate analysis failed to provide robust evidence suggesting the involvement of the OFC in reward anticipation and consummation. In the next section, we will investigate whether fine-grained multivariate activation patterns in OFC were more sensitive in revealing representations associated with reward values.

### Multivariate representational similarity fMRI analysis

In RSA, the primary data structure: representation dissimilarity matrix (RDM) is a correlation distance (1 – Pearson correlation) matrix of beta estimates associated with experimental conditions, reflecting brain activation patterns during tasks (e.g. anticipating a reward of ¥5.00) in concerned brain areas. Each element of the RDM is a correlation distance, called distance coefficient (DC), indicates similarity of activation patterns between the pairs of experimental conditions. A small DC indicates high similarity between multivariate brain activation patterns and vice versa.

### OFC encodes valence and magnitude of reward

During the anticipatory and consummatory phases, to examine whether the OFC encodes valence and magnitude information, we compared brain activation RDMs in the OFC with the a-priori model RDMs based on stimulus types (valence and magnitude) using Spearman’s correlation. Non-parametric permutation testing (10,000 permutations) was used to test the significance of correlations.

In the anticipatory phase before making a response, we found that activation RDM in the lOFC matched the simple model RDM for magnitude (specific, none vs. small + large) at a trend level (lOFC: *r* = 0.40, *p*_*permutation*_ = 0.07). In addition, the RDM of the mOFC showed a trend to match the simple and complex model RDM for magnitude (overall) (simple: *r* = 0.46, *p*_*permutation*_ = 0.07; complex: *r* = 0.45, *p*_*permutation*_ = 0.07). However, we did not observe any significant correlation between brain activation RDM in the OFC and model RDMs for valence (all *ps* > 0.1). In the anticipatory phase after making the response, there was a trend level correlation between the activation RDM in the lOFC and the simple model RDM for valence (loss) (lOFC: *r* = 0.46, *p*_*permutation*_ = 0.10) (See Table 1).

In the consummatory phase, similar to RDMs of the VS and the MPFC, RDMs of the mOFC and the lOFC significantly matched the simple and complex model RDMs for magnitude (overall) (*r* s = 0.23 to 0.54, *p*s_*permutation*_ < 0.05), suggesting that the OFC encoded magnitude information during the consummatory phase of reward processing. On the other hand, it was found that activation RDMs in the mOFC and the lOFC matched either the simple or complex model RDMs for valence (win vs. loss) (*r*s = 0.28 to 0.43, *p*s_*permutation*_ < 0.05), rather than model RDMs for outcome (*p*s_*permutation*_ > 0.1) (see Table 1), suggesting the OFC encoded valence (win vs. loss) regardless of favourable or unfavourable outcomes. The lOFC rather than the mOFC matched the simple model for loss (lOFC: *r* = 0.33, *p*_*permutation*_ = 0.004; mOFC: *r* = 0.02, *p*_*permutation*_ = 0.42).

### Comparing OFC with other regions within the reward network

In order to examine whether the mOFC and the lOFC encode value information similarly as the VS, the AI and the MPFC, we performed Spearman’s correlations between the RDMs at the mOFC and the lOFC, and RDM at the traditional reference regions during the anticipatory and consummatory phases.

We observed that the RDM of the OFC was similar to that of the VS (lOFC: *r* = 0.50, *p*_*permutation*_ = 0.03; but mOFC: *r* = 0.09, *p*_*permutation*_ = 0.37) and AI (lOFC: *r* = 0.47, *p*_*permutation*_ = 0.03; mOFC: *r* = 0.64, *p*_*permutation*_ = 0.01) in the anticipatory phase before making a response (See Fig. 4A). Within the win and loss components of the RDM, we did not observe any significant difference between the DCs of the mOFC, the lOFC, the VS, and the AI (*ps*_Bonferroni corrected_ > 0.1).

During the anticipatory phase after making the response, the RDMs of the lOFC were significantly similar to that of the VS (*r* = 0.66, *p*_*permutation*_ = 0.01) and show a trend level of similarity to that of the AI (*r* = 0.41, *p*_*permutation*_ = 0.099) (See Fig. 4A). Within the win or loss component RDM, there was no significant difference in DCs between the mOFC, the lOFC, the VS and the AI (*p*s_Bonferroni corrected_ > 0.1). Therefore, although no significant activation was detected on conventional univariate analysis, the RSA showed that the OFC was indeed encoding similar information as the VS/the AI during the anticipatory phase before and after making a response.

During the consummatory phase, RDMs of the mOFC and the lOFC were similar to those of the MPFC (mOFC: *r* = 0.79, *p*_*permutation*_ < 0.001; lOFC: *r* = 0.37, *p*_*permutation*_ = 0.001) and the VS (mOFC: *r* = 0.44, *p*_*permutation*_ = 0.001; lOFC: *r* = 0.68, *p*_*permutation*_ < 0.001), suggesting the involvement of the OFC in reward consummation (see Fig. 4B). For the win, loss avoidance, no win and loss components of the RDMs, there were no significant differences in DCs between the VS, the MPFC, the mOFC and the lOFC (*p*s_Bonferroni corrected_ > 0.1). Taken together, these findings suggest that the OFC was encoding similar information as the VS and the MPFC during reward consummation.

## Discussion

Our multivariate analysis showed that the OFC encoded magnitude and partial valence information during the consummatory phase of reward processing. The lOFC may represent loss information regardless of favourable or unfavourable outcome. During the anticipatory phase, the lOFC may encode magnitude information before making a response and valence information after making a response. In addition, the OFC exhibited similar activation patterns as the VS/AI during the anticipatory phase before and after making a response and as the MPFC and the VS during the consummatory phase. By contrast, OFC activations were not observed during the anticipatory or the consummatory phase using conventional univariate approaches suggesting that the neural representations of reward value in OFC maybe distributed across populations of neurons in human brains.

Although we did not find any OFC activation during the anticipatory or the consummatory phases of the MID task using conventional univariate analysis, the multi-voxel activation patterns in the OFC were largely similar to that of the VS/AI/MPFC using RSA. Our result is consistent with one previous study investigating value representation in the OFC during reward anticipation and consummation using multivariate pattern classification^23^. In this study, Kahnt found that the reward value of complicated sensory cues (i.e. rotation direction and colour) could be decoded from distributed fMRI patterns in the OFC during both reward anticipation and consummation^23^. They did not observe any significant activations using conventional analysis, either^23^. This suggests that multivariate pattern analysis may be a more sensitive and appropriate approach than conventional univariate analysis to detect neural responses in the OFC, which is a functionally and anatomically heterogeneous brain area^7^,^9^. Using a slightly different design (containing both reward and punishment cues and outcome) and different types of MVPA (i.e. RSA), we confirmed that multi-voxel fMRI pattern in the OFC encoded value during both reward anticipation and consummation, which is also consistent with evidences from animal studies^16^,^17^,^18^. In the present study, we investigated whether the OFC was involved in value processing during the anticipatory phase when no motivated response was required. It was found that the lOFC encoded values in a similar way as the AI and the VS during this phase. Although this result was less commonly reported in previous studies, it suggests that the OFC might be involved in value encoding during reward anticipation regardless of whether overt responses are involved or not.

During the consummatory phase, the OFC represented partial valence information (win vs. loss) regardless of outcome produced (favourable vs. unfavourable), which is consistent with previous findings suggesting that activities in the OFC may be associated with valence during anticipation^12^,^24^, decision making^35^,^36^ and consummation of outcome^37^,^38^. In the recent study using MVPA, Kahnt *et al*.^24^ found that multi-voxel patterns of the OFC represented valence information in the anticipatory phase. Specifically, the patterns coding for appetitive and aversive outcomes were similar, indicating a common neural circuit for encoding both appetitive and aversive values^24^. Extending the finding by Kanht and his colleagues, our study showed that OFC might also encode valence information in the consummatory phase. Furthermore, the lOFC rather than mOFC particularly responded to monetary loss, which is partly consistent with previous evidence suggesting that the medial-lateral OFC may differentially respond to rewarding and punishing events^13^,^14^. Unexpectedly, the way the OFC encoded valence was independent of outcome, possibly because that there are other connected brain regions that encode outcome information.

Interestingly, our findings suggest that the OFC also represents magnitude during the consummatory phase, which is different from previous studies suggesting that the OFC does not represent magnitude information^24^,^35^,^36^,^37^,^38^. By contrast, this is consistent with results from a previous meta-analysis, which reported that the mOFC/MPFC processes magnitude during reward consummation^34^. The inconsistency might be due to the paradigm selected in the studies. In the present study, the paradigm we employed yielded stronger multi-voxel patterns in the OFC during the consummatory phase. In most previous studies, participants were asked to either make a decision or to wait for the reward/punishment stimuli, which lacked the feeling of self-involvement and subjective effort^12^,^23^,^24^,^35^,^36^. However, in our MID task, outcomes were determined by explicit motor responses, which increased the sense of agency and motivation and might result in an arousal-like pattern in the OFC during the consummatory phase. Taken together, both valence and magnitude information appear to be encoded by the OFC during reward consummation.

During the anticipatory phase before participants’ making a response, the multi-voxel pattern of the mOFC and the lOFC tended to represent magnitude rather than valence information. This finding is similar to the VS showing increased activation for both anticipated gain and loss when outcomes were uncertain and salience was high^39^, supporting the anticipatory affect model proposed by Knutson and Greer^32^. In their model, the feeling of anticipation for future uncertain outcomes is closely related to human arousal regardless of valence^32^. A recent study has also suggested that the OFC may encode a general anticipatory value signal, regardless of reinforcer valence (appetitive/aversive)^40^. Interestingly, after the participants had responded, the lOFC tended to represent valence (especially for loss) instead, which is partly consistent with previous findings suggesting the OFC’s involvement in encoding valence^12^,^24^,^37^,^38^. The reason for the lOFC’s differential value representations during different anticipatory phase might be related to whether overt responses were required during the task. If motivated responses were not required, the OFC represents valence rather than magnitude of predicted outcomes^24^. Or, it might produce stronger multi-voxel patterns in the OFC encoding magnitude over valence information because overt response is closely related with arousal dimension of anticipatory affect^32^. Thus, our data suggests a possibility that multi-voxel pattern in the lOFC may differentially encode magnitude and valence information during the anticipatory phase of reward processing before and after making a response. Future research is needed to clarify the specific role of lOFC during reward anticipation.

This study has several limitations. First, the lOFC is an area where there might be relatively higher fMRI signal drop-out. To avoid signal drop-out in the lOFC due to magnetic susceptibility in homogeneity, we angled slices away from the orbits. Secondly, subjective ratings were obtained off-line, which might be noisy estimates of valence and arousal during the task due to the delay. Future studies should evaluate valence and arousal experience during the task.

## Conclusion

In conclusion, our findings suggest that similar to the VS, the AI and the MPFC, the OFC may also play an important role in value processing during both reward anticipation and consummation. The fine-grained multi-voxel activation pattern of the OFC might encode both valence and magnitude information in reward consummation.

## Methods

### Participants

Twenty-three participants (12 males; mean age: 19.78 (sd = 0.8)) were recruited from the East China Normal University and the Shanghai Normal University. All of them had no personal or family history of neurological, psychiatric or personality disorders. The study was approved by the Ethics Committee of the Institute of Psychology, the Chinese Academy of Sciences and was carried out in accordance with the approved guidelines. Written informed consent was obtained from all participants.

### Monetary Valence Delay Task

**Figure 1:**
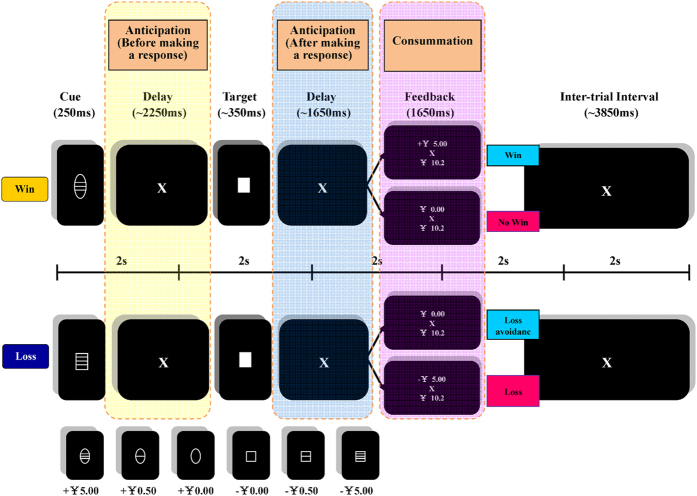
The scheme of the Monetary Valence Delay task. Each trial started with the presentation of a cue (circle/square), indicating the amount of money at stake (win or lose). The line inside the cue reflected the amount of money (no line =¥0, one line = ¥0.50 and three lines = ¥5.00). Following a pseudo-random delay (2000–2500 ms) in the anticipatory phase (before making a response), participants were required to respond to the target (a white solid square) by pressing the button as fast as possible using the right index finger. After a pseudo-random delay (1500–2500 ms) (anticipatory phase after making the response), a feedback (consummatory phase) was given to notify the participants about the amount of money they had won or lost as well as their cumulative balance.

We employed a modified version of the “Monetary Valence Delay Task” (MID) developed by Knutson *et al*.^31^. Each trial started with the presentation of a cue. The shapes of the cue (circle/square) indicated win or loss. The number of lines inside the cue reflected the amount of money (no line = ¥0, one line = ¥0.50 and three lines = ¥5.00). Following a pseudo-random delay (2000–2500 ms) in the anticipatory phase (before making a response), participants responded to the target (a white solid square) that appeared for a variable length of time (110 ms–560 ms) by pressing a button as quickly as possible with the right index finger. After another delay (anticipatory phase after making the response, 1500–2500 ms), a feedback was given to the participants indicating the amount of money they had won or lost and their current balance. Participants could receive or avoid loss of money by successfully pressing the button while the targets were still presented on the screen. Task difficulty, which was evaluated based on reaction times collected during the practice session before scanning, was matched across participants to have a success rate of approximately 66%. Each trial lasted for about 10 seconds (Fig. 1). Participants underwent three 9-minute-12-second sessions, each of which comprised 54 trials and were informed prior to the experiment that they would be paid with real money at the end of the experiment based on their winnings during the experiment. Participant earned between 23.7 (3.8 US$) to 56.7 (9.14 US$) RMB on average. The US Dollar to Chinese Yuan exchange rate was approximately 1 $ = ¥6.2 at the time of the experiment. In the MID task, three within-group variables were designed: valence (win vs. loss); magnitude (none (¥0) vs. small (¥0.50) vs. large (¥5.00)); and outcome (favourable vs. unfavourable. A favourable outcome refers to hitting the targets resulting in a non-zero reward or avoiding a non-zero loss).

After the scans, participants were immediately asked to rate their emotional state across the valence, magnitudes and outcome dimensions during the anticipatory and the consummatory phase of the MID task. A nine-point liker scale was used to measure valence (1 = extremely negative, 5 = neutral, 9 = extremely positive) and arousal (1 = extremely calm, 9 = extremely excited).

### Imaging acquisition

All the participants were scanned in a 3-Tesla Siemens Trio magnetic resonance imaging scanner (Siemens Medical Solutions, Erlangen, Germany). The functional images were acquired with the following sequence: TR = 2000 ms, TE = 30 ms, field of view (FOV) = 210 × 210 mm, flip angle = 90 degree, resulting in voxel size of 3.3 × 3.3 × 4 mm, Slice number = 32. To avoid signal drop-out in the lOFC due to magnetic susceptibility in homogeneity, we angled slices away from the orbits. Participants viewed visual stimuli on a projector screen via a mirror fixed on the head coil and responded with the right index finger by pressing the button on a response glove fixed on their right hand. High-resolution T1-weighted MPRAGE anatomy images were also acquired (TR = 2530 ms, TE = 30 ms, FOV = 256 × 256 mm, flip angle = 7 degree, 192 continuous axial slice of 1-mm thickness, voxel size = 1 × 1 × 1 mm).

### Data analysis

#### Behavioural data analysis

For motivated behaviour and subjective rating of anticipatory pleasantness, two-way (Valence × Magnitude) repeated measure ANOVAs were separately performed on reaction time, valence and arousal ratings. For the consummatory phase, a three-way (Outcome × Valence × Magnitude) repeated measure ANOVA was performed on valence and arousal. Valence, magnitude and outcome (favourable and unfavourable) were set as within-subject factors. Multiple comparisons were controlled using Bonferroni correction.

### fMRI data analysis

#### Pre-processing

Functional images were analysed using the SPM8 (Statistic Parametric Mapping, Welcome Department of Neurology, London, UK, 2009). Data pre-processing included realignment, slice timing, co-registration, anatomical segmentation and normalisation. Brain images were smoothed with 8 mm Gaussian kernel for univariate analysis but were unsmoothed for RSA in order to preserve the fine spatial details in the fMRI signal. For each participant, a general linear model (GLM) (Friston *et al*.^19^) was used to estimate a BOLD response for each condition containing 12 regressors for conditions during the anticipatory phase (-¥5.00, -¥0.50, -¥0.00, ¥0.00, ¥0.50, ¥5.00) before (6 regressors) and after (6 regressors) making a response, 12 regressors for different outcomes during the consummatory phase: win (expected reward), avoid loss (expected relief), no win (expected failure), loss (expected punishment) for three magnitudes, and six regressors for head movement parameters, a regressor for frame-wise displacement (FD)^41^, and separate regressors for each extreme spike (FD > 0.2 mm) across each run to control for head motion; one for whiter matter signal and one for cerebro-spinal fluid signal at the GLM analysis.

#### Conventional univariate analysis

The conventional univariate whole-brain analysis was performed using two-way repeated measure ANOVAs, which aimed to determine brain areas showing significant main effects of valence condition (win vs. loss), magnitude (large vs. small vs. none) and interactions of valence condition × magnitude in the anticipatory phase before and after making a response, respectively. For the consummatory phase, a three-way ANOVA analysis was performed with outcome (favourable vs. unfavourable), valence condition and magnitude as within-group variables. These contrasts were thresholded at *p* < 0.001 (uncorrected) at the peak level and *p* < 0.05 (Family Wise Error (FWE) for multiple comparison) at the cluster level, followed by small-volume correction.

### Representational Similarity Analysis

Independent from the univariate analysis, Representational Similarity Analysis (RSA)^27^,^28^,^29^,^30^ was carried out in anatomically defined ROIs. First, the brain RDMs were computed based on the beta estimates of each condition from the GLM using correlation distance (1 – Spearman’s rho). This resulted in a 6 × 6 (anticipatory) and a 12 × 12 (consummatory) brain RDM for each participant at the concerned ROIs in the anticipatory and the consummatory phase, respectively (see Fig. 4A,B). The value of the correlation distance, named distance coefficient (DC), indicates similarity of activation patterns between two conditions. For the ease of discussion, we will use the term “similarity” instead of “dissimilarity” in the rest of the paper.

**Figure 2:**
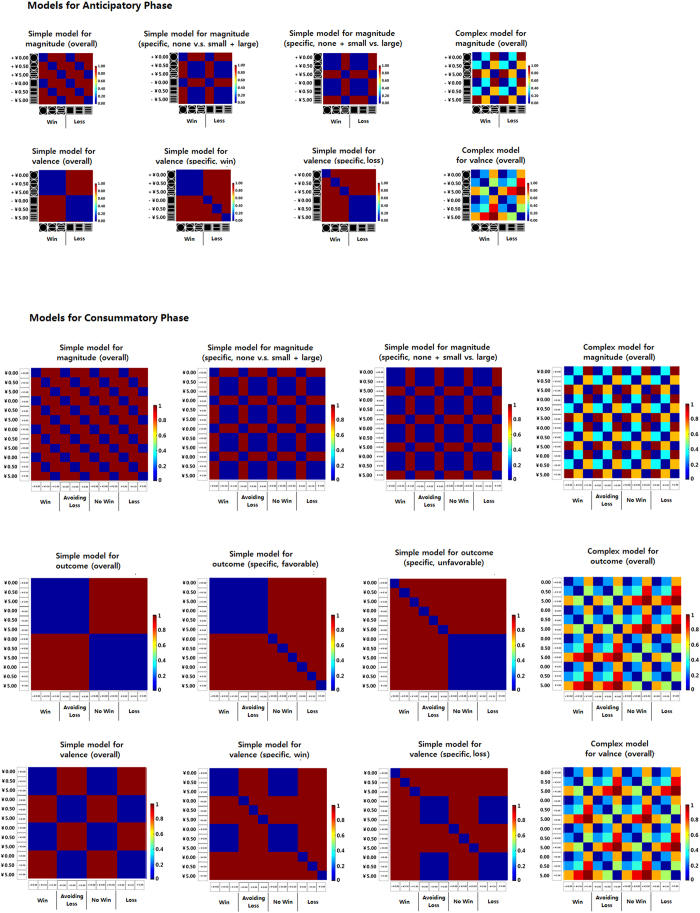
Model RDMs for anticipatory and consummatory phase. **(A**) For anticipatory phase, we had three types of models for magnitude regardless of valence, including the simple model for magnitude (overall), the simple model for magnitude (specific, none vs. small + large)/the simple model for magnitude (specific, none + small vs. large), and the complex model for magnitude (overall). We also had three types of models for valence without considering magnitude, including the simple model for valence (overall), the simple model for valence (specific, win)/the simple model for valence (specific, loss), and the complex model for valence (overall). (**B**) For consummatory phase, we had three types of models for magnitude without considering valence and outcome and four models for valence regardless of outcome and magnitude. In addition, we had three types of models for outcome regardless of valence and magnitude, including the simple model for outcome (overall), the simple model for outcome (specific, favorable)/the simple model for outcome (specific, unfavorable), and the complex model for outcome (overall). Blue indicates that the pattern between two conditions is same (DC = 0), while the color brown indicates that the patterns is different (DC = 1). In the simple model, the relationships between conditions were completely the same or different (DC = 0 or 1). In the complex model, the relationships between conditions were relatively the same or different (DC ranged from 0 to 1).

**Figure 3:**
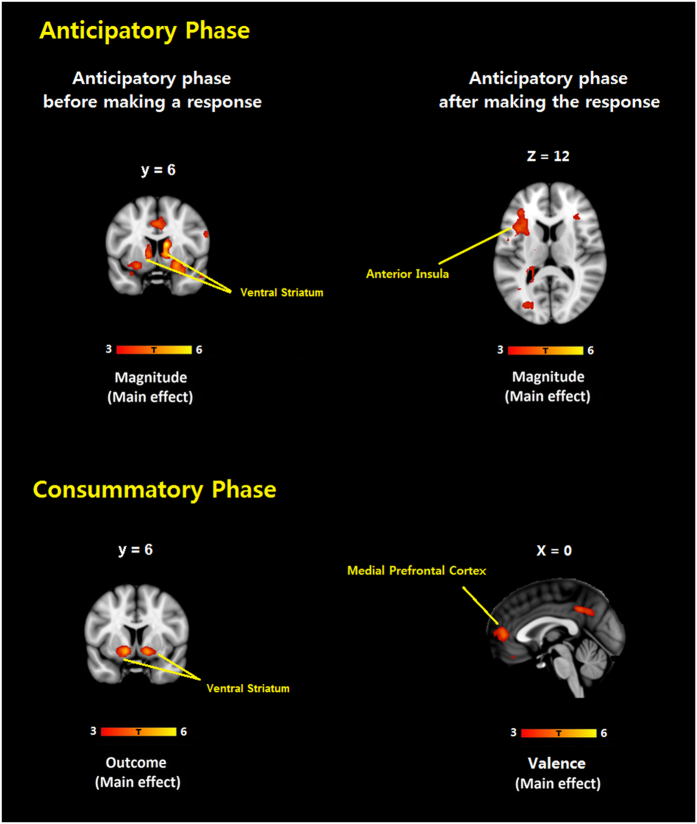
Whole brain activations during anticipatory phase and consummatory phase. Multivariate comparison was accomplished using Family Wise Error (FWE) correction (*p* < 0.05) at cluster level. Clusters in brighter colour represent stronger activation.

**Figure 4:**
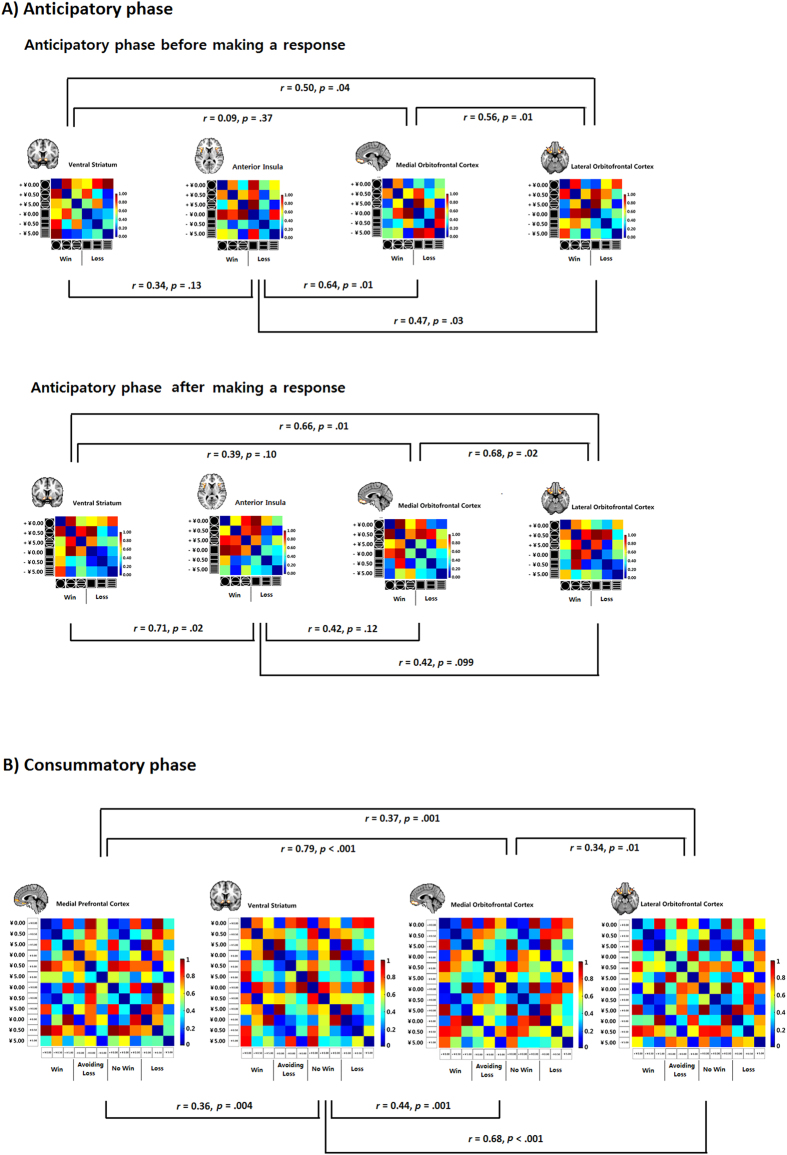
Multi-voxel patterns of the mOFC, and the lOFC during the anticipatory and the consummatory phase. RDMs for anticipatory phase (before and after making a response) in the mOFC, the lOFC, the VS and the AI are shown on the upper panel (**4A**). Graphs on the bottom panel represent RDMs for consummatory phase in the mOFC, the lOFC, the VS and the MPFC (**4B**). Each anticipatory and consummatory RDMs separately rank transformed and scaled into [0, 1]. Relationships between anticipatory and consummatory RDMs between all the ROIs were marked under the RDM.

To detect whether the mOFC and the lOFC represented valence and magnitude information in the anticipatory and the consummatory phases, we defined a number of model RDMs which reflected valence and magnitude coding, and used Spearman correlations to test whether brain RDMs of the mOFC and the lOFC matched these a-priori model RDMs. There were three types of model RDMs for each type of value information (valence, magnitude, outcome): a simple model for overall value encoding (i.e. valence; DC = 0 or 1; where 0 indicates that the two conditions are the same while 1 indicates the difference); simple models for specific value encoding (i.e. win and loss for the valence model, respectively) and complex models for overall value encoding in a transitional way (i.e. valence, DC ranged from 0 to 1). The simple model reflects that the value was encoded as “all or none” (either the same or different between conditions) while complex models represent a gradual difference between values. For example, within the model RDM for valence, winning large reward was assumed to be completely the same as winning no reward in the simple model (DC = 0), but partly similar to winning no reward (DC = 0.5) in the complex model (please see supplementary material for more details). Then we performed the Spearman’s correlation between the brain activation patterns in the OFC and the models reflecting different value representations. (see Fig. 2 and the Supplementary Materials for more details).

Non-parametric permutation testing (10,000 permutations) was used to see whether multi-voxel patterns in the OFC were similar to the patterns in the traditional reference regions such as VS, the AI and the MPFC as reported by previous researches^4^,^32^,^34^. Spearman correlations were used to test the correlations between RDMs of the mOFC, lOFC, VS and AI in the anticipatory phase and correlations between the RDMs of the mOFC, lOFC, VS and MPFC in the consummatory phase, respectively.

Within the anticipatory/consummatory phase, we further examined whether the patterns of the sub-components of the RDM (including “Win” and “Loss” component within the anticipatory RDM, and “Win”, “Avoid Loss”, “No Win”, and “Loss” component within the consummatory RDM, Supplementary Fig. 2) were different or not between ROIs. Standardized Fisher-transformed DCs within each sub-component of RDM were extracted and averaged across three runs. Independent t-tests were performed on averaged DCs of subcomponents between different ROIs. P value was adjusted using Bonferroni correction.

### ROIs selection

All regions of interest (ROI) were anatomically defined based on brain atlases^42^ implemented in Wake Forest University PickAtlas toolbox (version 3.0 4) in the SPM 8^43^. Because of previous meta-analysis implicating the VS and the AI in the value processing during anticipatory phase^4^,^32^ and the VS and the MPFC during consummatory phase^32^,^34^, we considered these regions as traditional regions associated with value processing. The mOFC and the lOFC, on the other hand, was the ROIs for which we hypothesized based on previous studies^7^,^8^,^10^,^23^,^40^. We defined anatomical ROI masks including a MPFC mask (BA 10, BA 32 and BA 25)^44^, a AI mask (BA 13 bounded caudally at y = 0 to include only the anterior region)^45^,^46^, a mOFC mask (BA 11)^44^ and a lOFC mask (BA 12 and BA 47)^7^ based on Brodmann Areas Map as well as a VS mask (nucleus accumbens) based on Individual Brain Atlases using Statistical Parametrical Mapping (IBASPM)^32^. All of these ROIs were defined in MNI space.

## Additional Information

**How to cite this article**: Yan, C. *et al*. Multivariate Neural Representations of Value during Reward Anticipation and Consummation in the Human Orbitofrontal Cortex. *Sci. Rep.* **6**, 29079; doi: 10.1038/srep29079 (2016).

## Supplementary Material

Supplementary Information

## Figures and Tables

**Table 1:** Relationships between Brain RDM in the mOFC, lOFC and Model RDMs for anticipatory and consummatory phase.

	mOFC	lOFC	VS	AI	MPFC
Model RDM for Anticipatory Phase before Making a Response					
Simple model for magnitude (overall)	0.46^†^	0.39	0.31	0.27	–
Simple model for magnitude (specific, none vs. small + large)	0.12	0.40^†^	0.43	0.34	–
Simple model for magnitude (specific, none + small vs. large)	0.43	0.06	−0.06	0.19	–
Complex model for magnitude (overall)	0.45^†^	0.23	0.13	0.33	–
Simple model for valence (overall)	−0.16	0.19	0.09	0.06	–
Simple model for valence (specific, win)	0.08	−0.08	−0.31	−0.08	–
Simple model for valence (specific, loss)	−0.27	0.31	0.42	0.15	–
Complex model for valence (overall)	0.01	−0.17	−0.36	−0.25	–
Model RDM for Anticipatory Phase after Making a Response					
Simple model for magnitude (overall)	−0.42	−0.15	0.12	−0.04	–
Simple model for magnitude (specific, none vs. small + large)	−0.09	0.22	0.34	0.34	–
Simple model for magnitude (specific, none + small vs. large)	−0.53	−0.15	−0.15	−0.25	–
Complex model for magnitude (overall)	−0.53	−0.04	0.01	−0.07	–
Simple model for valence (overall)	−0.06	0.09	0.16	−0.03	–
Simple model for valence (specific, win)	−0.42	−0.35	−0.42	−0.39	–
Simple model for valence (specific, loss)	0.35	0.46^†^	0.62^†^	0.35	–
Complex model for valence (overall)	−0.41	−0.15	−0.29	−0.45	–
Model RDM for Consummatory Phase					
Simple model for magnitude (overall)	0.41^**^	0.23^*^	0.51^**^	–	0.45^**^
Simple model for magnitude (specific, none vs. small + large)	0.48^**^	0.13	0.50^**^	–	0.38^*^
Simple model for magnitude (specific, none + small vs. large)	0.38^*^	0.03	0.29^*^	–	0.29^*^
Complex model for magnitude (overall)	0.54^**^	0.09	0.48^**^	–	0.43^**^
Simple model for outcome (overall)	−0.13	−0.12	−0.11	–	−0.12
Simple model for outcome (specific, favorable)	−0.02	−0.13	−0.07	–	−0.06
Simple model for outcome (specific, unfavorable)	−0.13	−0.01	−0.07	–	−0.08
Complex model for outcome (overall)	0.13	−0.01	0.16^†^	–	0.12
Simple model for valence (overall)	0.16	0.43^**^	0.24^*^	–	0.25^†^
Simple model for valence (specific, win)	0.17	0.18^†^	−0.14	–	0.24^†^
Simple model for valence (specific, loss)	0.02	0.33^**^	0.42^**^	–	0.06
Complex model for valence (overall)	0.28^*^	0.19^†^	0.26^**^	–	0.34^**^

Note: *p < 0.05; **p < 0.01; ^†^0.05 < *p* < 0.1. Significance was assessed using non-parametric permutation testing. mOFC = medial orbitofrontal cortex, lOFC = lateral orbitofrontal cortex, VS = ventral striatum, AI = anterior insular, MPFC = medial prefrontal cortex, RDM = representational dissimilarity matrix.
