# Supplementary material for: Multivariate Neural Representations of Value during Reward Anticipation and Consummation in the Human Orbitofrontal Cortex
Source: Sci Rep. 2016 Jul 5;6:29079. doi: 10.1038/srep29079 (PMC4932626; doi:10.1038/srep29079)
Supplement: Supplementary Information [file srep29079-s1.doc]

**Multivariate Neural Representations of Value during Reward Anticipation and Consummation in the Human Orbitofrontal Cortex**

Chao Yan^1,2^, Li Su^3^, Yi Wang^1^, Ting Xu^1^, Da-zhi Yin^4^, Ming-xia Fan^5^, Ci-ping Deng^2^, Yang Hu^2^, Zhao-xin Wang^2^, Eric F. C. Cheung^6^, Kelvin O. Lim^7^, Raymond C. K. Chan^1^*

1: Neuropsychology and Applied Cognitive Neuroscience Laboratory, Key Laboratory of Mental Health, Institute of Psychology, Chinese Academy of Sciences, Room 606, South Building, 16 Lincui Road, Beijing, Beijing, China, 100101

2: Key Laboratory of Brain Functional Genomics, Ministry of Education, Shanghai Key Laboratory of Brain Functional Genomics (MOE & STCSM), East China Normal University, Room 212, Junxiu Building, 3663 North Zhongshan Road, Shanghai, China, 200062

3: Department of Psychiatry, Cambridge Biomedical Campus, University of Cambridge, Cambridge，UK, CB2 0SP

4: Institute of Neuroscience, Shanghai Institutes for Biological Sciences, Chinese Academy of Sciences, 320 Yue Yang Road, Shanghai, China, 200031

5: Shanghai Key Laboratory of MRI, East China Normal University, 3663 North Zhongshan Road, Shanghai, China, 200062

6: Department of General Adult Psychiatry, Castle Peak Hospital, 15 Tsing Chung Koon Road, Tuen Mun, N.T. Hong Kong Special Administrative Region, China

7: Department of Psychiatry, University of Minnesota, F282/2A West 2450 Riverside Avenue, Minneapolis, USA, MN 55454

*corresponding author:

Raymond CK Chan, Room 526, South Building, Institute of Psychology, Chinese Academy of Sciences, 16 Lincui Road, Beijing, China; Tel/Fax: 86(10)64836274

e-mail: [rckchan@psych.ac.cn](mailto:rckchan@psych.ac.cn)

**Supplementary Materials**

**
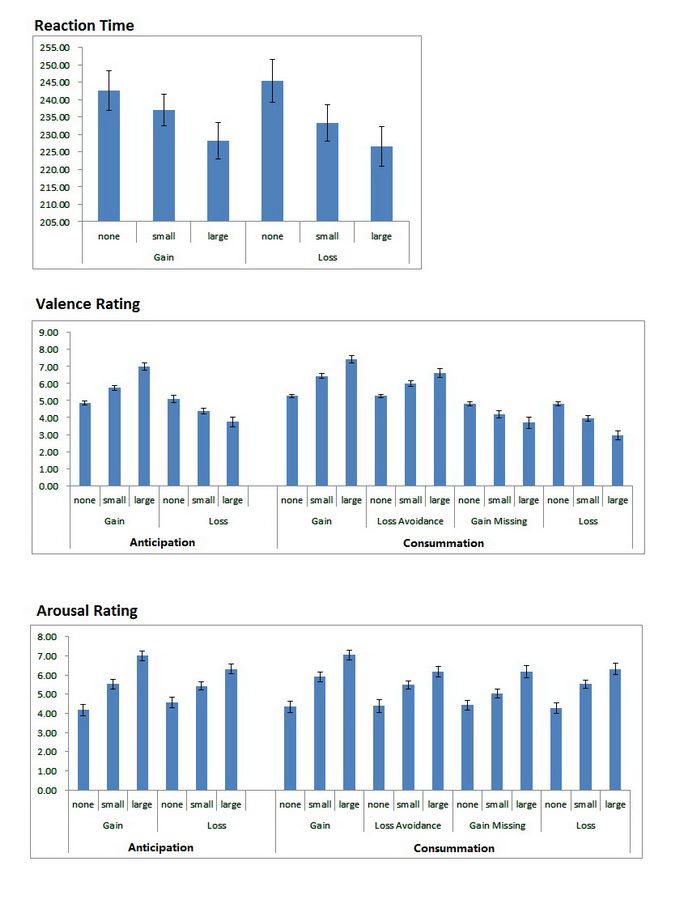
**

**Supplementary Fig.1. Behavioral data for MID task.** The blue bar graphs on the top panel represent the motivated behaviour (milliseconce). Low value indicates that participants responded fast to the target. The bars on the middle panel represent subjectively valence rating of anticipatory and consummatory affect. High value (> 5) indicates the pleasant experience while low value (< 5) indicates the aversive experience. The bars on the bottom panel represent subjectively arousal rating of anticipatory and consummatory affect. High value (> 5) indicates the exciting feeling whereas low value (< 5) indicates the calm feeling.

**Supplementary Table 1.** Whole brain activations in anticipatory phase before making a response.

|  | **Cluster**  **size** | **T/F**  **statistic** | **Z**  **statistic** | **x** | **y** | **z** | **Brain Regions** | **BA** |
| --- | --- | --- | --- | --- | --- | --- | --- | --- |
| **Main Effect**  **(Valence, win vs. loss)** | n.s. |  |  |  |  |  |  |  |
|  |  |  |  |  |  |  |  |  |
| **Main Effect**  **(Magnitude, large vs. small vs. none)** | 259 | 7 | 6.44 | 12 | 0 | 15 | R. Caudate body  (extending to VS) |  |
|  |  | 5.38 | 5.1 | 12 | 6 | 3 |  |  |
|  |  | 4.85 | 4.64 | 0 | -6 | 15 |  |  |
|  | 227 | 5.83 | 5.49 | -24 | 0 | -12 | L. Putamen |  |
|  |  | 4.15 | 4.01 | -33 | -12 | -15 |  |  |
|  |  | 4.14 | 4.01 | -33 | -18 | -9 |  |  |
|  | 162 | 5.58 | 5.28 | 27 | 0 | -12 | R. Putamen |  |
|  |  | 4.33 | 4.18 | 30 | 9 | -21 |  |  |
|  |  | 3.72 | 3.62 | 30 | -15 | -9 |  |  |
|  | 696 | 5.47 | 5.18 | -12 | -15 | 42 | dACC | 24 |
|  |  | 5.19 | 4.94 | 0 | -12 | 39 |  |  |
|  |  | 5.02 | 4.8 | 6 | -18 | 42 |  |  |
|  | 233 | 4.47 | 4.31 | -12 | -27 | -30 | Culmen |  |
|  |  | 4.42 | 4.26 | 15 | -42 | -21 |  |  |
|  |  | 4.36 | 4.20 | 3 | -45 | -18 |  |  |
|  |  |  |  |  |  |  |  |  |
| **Interaction Effect** | n.s. |  |  |  |  |  |  |  |
| **(Valence X Magnitude)** |  |  |  |  |  |  |  |  |

***Note:*** reported are the results for the two-way (valence x magnitude) repeated ANOVA of anticipation before making responses with Brodmann area (BA), cluster size, T/F statistic, z statistic, and MNI coordinate for the whole brain. The contrasts were thresholded at p < .05 (FWE cluster voxel corrected). R. = Right; L. = Left. VS = Ventral Striatum; dACC = dorsal anterior cingulate cortex.

**Supplementary Table 2.** Whole brain activations in anticipatory phase after making a response.

|  | **Cluster**  **size** | **T/F**  **statistic** | **Z**  **statistic** | **x** | **y** | **z** | **Brain Regions** | **BA** |
| --- | --- | --- | --- | --- | --- | --- | --- | --- |
| **Main Effect**  **(Valence, win vs. loss)** | n.s. |  |  |  |  |  |  |  |
|  |  |  |  |  |  |  |  |  |
| **Main Effect**  **(Magnitude, large vs. small vs. none)** | 157 | 4.51 | 4.34 | -27 | -42 | 6 | L. Hippocampus |  |
|  |  | 4.25 | 4.10 | -33 | -48 | 6 |  |  |
|  |  | 3.75 | 3.65 | -35 | -60 | 9 |  |  |
|  | 88 | 4.08 | 3.95 | -36 | 15 | 12 | L. AI | 13 |
|  |  | 3.92 | 3.81 | -36 | 33 | 12 |  |  |
|  |  | 3.50 | 3.42 | -30 | 21 | 0 |  |  |
|  |  |  |  |  |  |  |  |  |
| **Interaction Effect**  **(Valence X Magnitude)** | n.s |  |  |  |  |  |  |  |

***Note:*** reported are the results for the two-way (valence x magnitude) repeated ANOVA of anticipation after having made responses with Brodmann area (BA), cluster size, T/F statistic, z statistic, and MNI coordinate for the whole brain. The contrasts were thresholded at p < .05 (FWE cluster voxel corrected). R. = Right; L. = Left. AI = Anterior Insula.

**Supplementary Table 3.** Whole brain activations during consummatory phase.

|  | **Cluster**  **size** | **T/F**  **statistic** | **Z**  **statistic** | **x** | **y** | **z** | **Brain Regions** | **BA** |
| --- | --- | --- | --- | --- | --- | --- | --- | --- |
| **Main Effect (Outcome)**  **(favorable vs. unfavorable)** | 94 | 6.08 | 5.88 | -12 | 6 | -9 | L. LGP  (extending to VS) |  |
|  |  |  |  |  |  |  |  |  |
| **Main Effect (Valence)**  **(win vs. loss)** | 241 | 5.49 | 5.34 | -21 | -60 | 24 | Posterior ACC | 31 |
|  |  | 4.94 | 4.83 | -12 | -57 | 24 |  |  |
|  |  | 3.97 | 3.91 | 0 | -45 | 39 |  |  |
|  | 126 | 4.38 | 4.30 | 0 | 48 | 6 | MPFC | 9 |
|  |  |  |  | 6 | 60 | 3 |  |  |
|  |  |  |  |  |  |  |  |  |
| **Main Effect (Magnitude)**  **(large vs. small vs. none)** | n.s. |  |  |  |  |  |  |  |
|  |  |  |  |  |  |  |  |  |
| **Interaction Effect**  **(Outcome x Valence x Magnitude)** | 59 | 24.13 | 4.55 | -54 | -51 | 27 | L. SG | 40 |
|  |  | 20.17 | 4.17 | -53 | -48 | 30 |  |  |
|  |  | 17.89 | 3.92 | -48 | -57 | 27 |  |  |
|  |  |  |  |  |  |  |  |  |
| **Interaction Effect**  **(Outcome X Valence)** | n.s. |  |  |  |  |  |  |  |
|  |  |  |  |  |  |  |  |  |
| **Interaction Effect**  **(Outcome X Magnitude)** | n.s. |  |  |  |  |  |  |  |
|  |  |  |  |  |  |  |  |  |
| **Interaction Effect**  **(Outcome X Magnitude)** | n.s. |  |  |  |  |  |  |  |

***Note:*** reported are the results for the three-way repeated ANOVA of consummatory phaase with Brodmann area (BA), cluster size, T/F statistic, z statistic, and MNI coordinate for the whole brain. The contrasts were thresholded at p < .05 (FWE cluster voxel corrected). R. = Right; L. = Left. VS = Ventral Striatum; LGP = Lateral Globus Pallidus; MPFC = Medial Prefrontal Cortex; ACC = Anterior Cingulate Cortex; SG = Supermarginal Gyrus.

**Univariate ROI analysis**

In order to further explore the univariate activation in the OFC, we performed three-way (valence x magnitude x regions) and four-way (outcome x valence x magnitude x regions) repeated measures ANOVAs to separately test whether BOLD signal change percentage in the mOFC/lOFC were different from those in the VS/AI during the anticipatory phases and whether they were different from those in the VS/MPFC during the consummatory phase.

Supplementary Fig.2A shows that the BOLD signal changes in the mOFC and the lOFC in the anticipatory phase before making a response were indeed significantly weaker than those in the VS, reflected by a main effect for region (F (2, 51) _Greenhouse-Geisser adjusted_ = 6.579, p = .002; VS > mOFC, *p* < .001; VS > lOFC, *p* = .03).

During the anticipatory phase after making the response, there was a significant main effect for region (F (3,56) _Greenhouse-Geisser adjusted_ = 6.436, p = .001), indicating weaker activation in the mOFC and lOFC compared to the AI (AI > mOFC, *p* = .008; AI > lOFC, *p* = .009). (See Supplementary Fig.2A).

During the consummatory phase, we observed that the lOFC and the mOFC did not activate more for favourable outcomes compared to unfavourable outcomes (lOFC: F (1,22) = 0.47, p = .50; mOFC: F (1,22) = 2.96, p = .10), which was different from the activities in the VS and the MPFC (Favourable > Unfavourable: VS: F (1,22) = 4.37, p = .048; MPFC: F (1,22) = 5.42, p = .03), reflected by a significant interaction effect for regions x outcomes (*F* (2,27) _Greenhouse-Geisser adjusted_ = 4.529, *p* = .035). (See Supplementary Fig.2B).


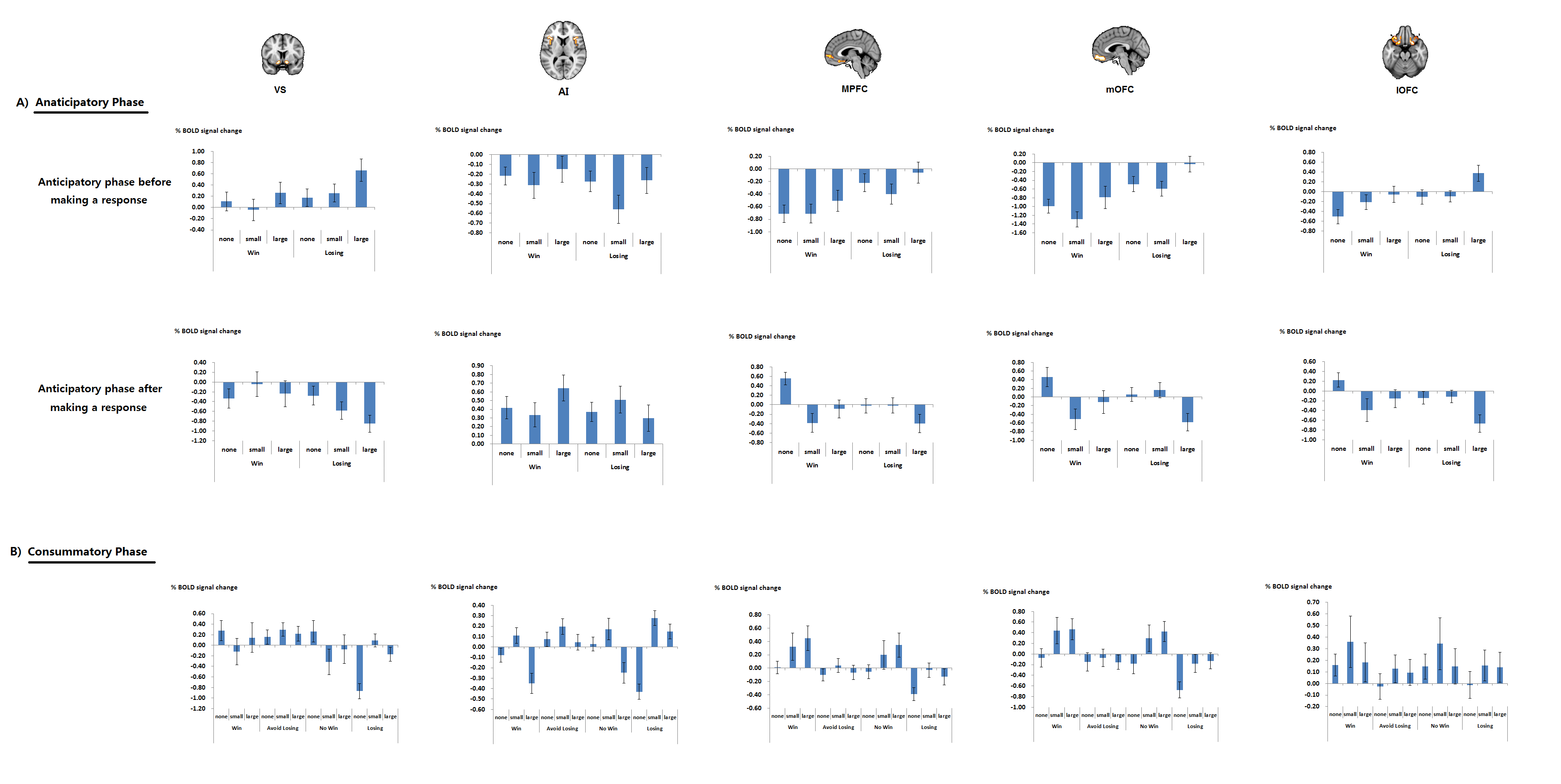


**Supplementary Fig.2 Univariate activation during anticipatory and consummatory phase.** BOLD signal change percentages for anticipatory phase (before and after making a response) in the mOFC, the lOFC, the VS, the AI and the MPFC are shown on the upper panel (A). Graphs on the bottom panel represent BOLD signal change percentages for consummatory phase in the mOFC, the lOFC, the VS, the AI and the MPFC (B).

**Supplementary Fig.3 Sub-components of RDMs during anticipatory and consummatory phase.** Anticipatory phase: 1 = “Win” sub-component, 2 = “Loss” sub-component, Consummatory phase: 3 = “Win” sub-component, 4 = “Avoid Loss” sub-component, 5 = “No Win” sub-component, 6 = “Loss” sub-component.


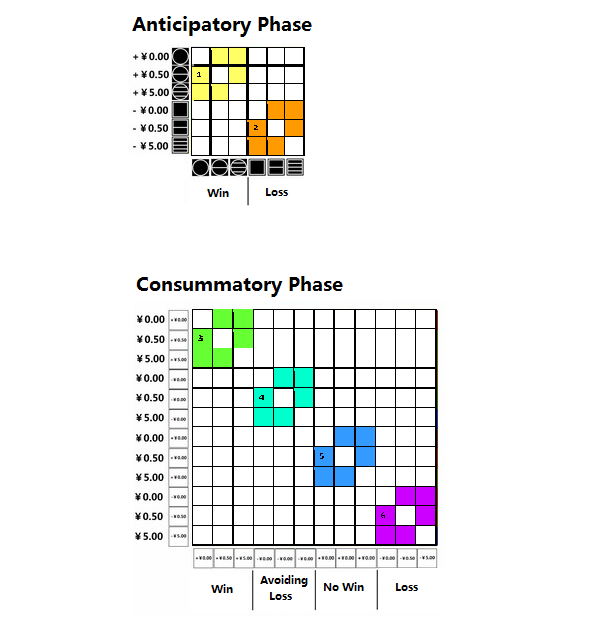


**Model RDMs setting for anticipatory and consummatory phase**

For anticipatory phase, we had three types of models for magnitude and valence: simple model for magnitude/valence (overall), simple model for magnitude/valence (specific), and complex model for magnitude/valence (overall). Within the simple model for magnitude/valence (specific), there were further two models reflecting specific value encoding (i.e. win for valence model) within the model for valence or magnitude. For the simple model for magnitude (specific), we had two specific models (non-reward vs. reward (small + large) and non-large reward (none + small) vs. large) instead of three models (none, small, large) because there were fewer parameters for the three specific model to estimation, which would lead to unreliable model computation. In the model for magnitude (simple), we defined that brain patterns of the none, small, large magnitude were completely different from each other (DCs = 1), regardless of win or loss condition. Simple model reflects that the value was encoded as “all or none” (completely same or different) while complex model reflects a transitional way to encode value (relatively similar or different). For example, within the model RDM for valence, winning large reward was assumed to be completely the same as winning none reward in the simple model (DC = 0), but partly similar to winning non reward (DC = 0.5) in the complex model . (see the details for settings of each model below).

**Simple Models for Magnitude (overall**)

|  |  |  | Win |  |  | Loss |  |
| --- | --- | --- | --- | --- | --- | --- | --- |
|  |  | none | small | large | none | small | large |
| Win | none | 0 | 1 | 1 | 0 | 1 | 1 |
|  | small | 1 | 0 | 1 | 1 | 0 | 1 |
|  | large | 1 | 1 | 0 | 1 | 1 | 0 |
| Loss | none | 0 | 1 | 1 | 0 | 1 | 1 |
|  | small | 1 | 0 | 1 | 1 | 0 | 1 |
|  | large | 1 | 1 | 0 | 1 | 1 | 0 |

**Simple Models for Magnitude (specific, none vs. small + large)**

|  |  |  | Win |  |  | Loss |  |
| --- | --- | --- | --- | --- | --- | --- | --- |
|  |  | none | small | large | none | small | large |
| Win | none | 0 | 1 | 1 | 0 | 1 | 1 |
|  | small | 1 | 0 | 0 | 1 | 0 | 0 |
|  | large | 1 | 0 | 0 | 1 | 0 | 0 |
| Loss | none | 0 | 1 | 1 | 0 | 1 | 1 |
|  | small | 1 | 0 | 0 | 1 | 0 | 0 |
|  | large | 1 | 0 | 0 | 1 | 0 | 0 |

**Simple Models for Magnitude (specific, none + small vs. large)**

|  |  |  | Win |  |  | Loss |  |
| --- | --- | --- | --- | --- | --- | --- | --- |
|  |  | none | small | large | none | small | large |
| Win | none | 0 | 0 | 1 | 0 | 0 | 1 |
|  | small | 0 | 0 | 1 | 0 | 0 | 1 |
|  | large | 1 | 1 | 0 | 1 | 1 | 0 |
| Loss | none | 0 | 0 | 1 | 0 | 0 | 1 |
|  | small | 0 | 0 | 1 | 0 | 0 | 1 |
|  | large | 1 | 1 | 0 | 1 | 1 | 0 |

**Complex Models for Magnitude (overall)**

|  |  |  | Win |  |  | Loss |  |
| --- | --- | --- | --- | --- | --- | --- | --- |
|  |  | none | small | large | none | small | large |
| Win | none | 0 | 0.05 | 0.5 | 0 | 0.05 | 0.5 |
|  | small | 0.05 | 0 | 0.45 | 0.05 | 0 | 0.45 |
|  | large | 0.5 | 0.45 | 0 | 0.5 | 0.45 | 0 |
| Loss | none | 0 | 0.05 | 0.5 | 0 | 0.05 | 0.5 |
|  | small | 0.05 | 0 | 0.45 | 0.05 | 0 | 0.45 |
|  | large | 0.5 | 0.45 | 0 | 0.5 | 0.45 | 0 |

**Simple Models for Valence (overall)**

|  |  |  | Win |  |  | Loss |  |
| --- | --- | --- | --- | --- | --- | --- | --- |
|  |  | none | small | large | none | small | large |
| Win | none | 0 | 0 | 0 | 1 | 1 | 1 |
|  | small | 0 | 0 | 0 | 1 | 1 | 1 |
|  | large | 0 | 0 | 0 | 1 | 1 | 1 |
| Loss | none | 1 | 1 | 1 | 0 | 0 | 0 |
|  | small | 1 | 1 | 1 | 0 | 0 | 0 |
|  | large | 1 | 1 | 1 | 0 | 0 | 0 |

**Simple Models for Valence (specific, win)**

|  |  |  | Win |  |  | Loss |  |
| --- | --- | --- | --- | --- | --- | --- | --- |
|  |  | none | small | large | none | small | large |
| Win | none | 0 | 0 | 0 | 1 | 1 | 1 |
|  | small | 0 | 0 | 0 | 1 | 1 | 1 |
|  | large | 0 | 0 | 0 | 1 | 1 | 1 |
| Loss | none | 1 | 1 | 1 | 0 | 1 | 1 |
|  | small | 1 | 1 | 1 | 1 | 0 | 1 |
|  | large | 1 | 1 | 1 | 1 | 1 | 0 |

**Simple Models for Valence (specific, loss)**

|  |  |  | Win |  |  | Loss |  |
| --- | --- | --- | --- | --- | --- | --- | --- |
|  |  | none | small | large | none | small | large |
| Win | none | 0 | 1 | 1 | 1 | 1 | 1 |
|  | small | 1 | 0 | 1 | 1 | 1 | 1 |
|  | large | 1 | 1 | 0 | 1 | 1 | 1 |
| Loss | none | 1 | 1 | 1 | 0 | 0 | 0 |
|  | small | 1 | 1 | 1 | 0 | 0 | 0 |
|  | large | 1 | 1 | 1 | 0 | 0 | 0 |

**Complex Models for Valence (overall)**

|  |  |  | Win |  |  | Loss |  |
| --- | --- | --- | --- | --- | --- | --- | --- |
|  |  | none | small | large | none | small | large |
| Win | none | 0 | 0.05 | 0.5 | 0 | 0.05 | 0.5 |
|  | small | 0.05 | 0 | 0.45 | 0.05 | 0.1 | 0.55 |
|  | large | 0.5 | 0.45 | 0 | 0.5 | 0.55 | 1 |
| Loss | none | 0 | 0.05 | 0.5 | 0 | 0.05 | 0.5 |
|  | small | 0.05 | 0.1 | 0.55 | 0.05 | 0 | 0.45 |
|  | large | 0.5 | 0.55 | 1 | 0.5 | 0.45 | 0 |

For consummatory phase, there was another model RDM for outcome (favorable (win + avoid loss) vs. unfavorable (no win + loss)). Like the model setting in the anticipatory phase, we have three types of model: simple model (overall), simple model (specific) and complex model (overall) (see the details for settings of each model below).

**Simple Models for Magnitude (overall)**

|  |  |  | Favorable Outcomes | | | | | | Unfavorable Outcomes | | | | | |
| --- | --- | --- | --- | --- | --- | --- | --- | --- | --- | --- | --- | --- | --- | --- |
|  |  |  | Win | | | Loss | | | Win | | | Loss | | |
|  |  |  | none | small | large | none | small | large | none | small | large | none | small | large |
| Favorable  Outcomes | Win | none | 0 | 1 | 1 | 0 | 1 | 1 | 0 | 1 | 1 | 0 | 1 | 1 |
|  |  | small | 1 | 0 | 1 | 1 | 0 | 1 | 1 | 0 | 1 | 1 | 0 | 1 |
|  |  | large | 1 | 1 | 0 | 1 | 1 | 0 | 1 | 1 | 0 | 1 | 1 | 0 |
|  | Loss | none | 0 | 1 | 1 | 0 | 1 | 1 | 0 | 1 | 1 | 0 | 1 | 1 |
|  |  | small | 1 | 0 | 1 | 1 | 0 | 1 | 1 | 0 | 1 | 1 | 0 | 1 |
|  |  | large | 1 | 1 | 0 | 1 | 1 | 0 | 1 | 1 | 0 | 1 | 1 | 0 |
| Unfavorable  outcomes | Win | none | 0 | 1 | 1 | 0 | 1 | 1 | 0 | 1 | 1 | 0 | 1 | 1 |
|  |  | small | 1 | 0 | 1 | 1 | 0 | 1 | 1 | 0 | 1 | 1 | 0 | 1 |
|  |  | large | 1 | 1 | 0 | 1 | 1 | 0 | 1 | 1 | 0 | 1 | 1 | 0 |
|  | Loss | none | 0 | 1 | 1 | 0 | 1 | 1 | 0 | 1 | 1 | 0 | 1 | 1 |
|  |  | small | 1 | 0 | 1 | 1 | 0 | 1 | 1 | 0 | 1 | 1 | 0 | 1 |
|  |  | large | 1 | 1 | 0 | 1 | 1 | 0 | 1 | 1 | 0 | 1 | 1 | 0 |

**Simple Models for Magnitude (specific**, none vs. small + large)

|  |  |  | Favorable Outcomes | | | | | | Unfavorable Outcomes | | | | | |
| --- | --- | --- | --- | --- | --- | --- | --- | --- | --- | --- | --- | --- | --- | --- |
|  |  |  | Win | | | Loss | | | Win | | | Loss | | |
|  |  |  | none | small | large | none | small | large | none | small | large | none | small | large |
| Favorable  Outcomes | Win | none | 0 | 1 | 1 | 0 | 1 | 1 | 0 | 1 | 1 | 0 | 1 | 1 |
|  |  | small | 1 | 0 | 0 | 1 | 0 | 0 | 1 | 0 | 0 | 1 | 0 | 0 |
|  |  | large | 1 | 0 | 0 | 1 | 0 | 0 | 1 | 0 | 0 | 1 | 0 | 0 |
|  | Loss | none | 0 | 1 | 1 | 0 | 1 | 1 | 0 | 1 | 1 | 0 | 1 | 1 |
|  |  | small | 1 | 0 | 0 | 1 | 0 | 0 | 1 | 0 | 0 | 1 | 0 | 0 |
|  |  | large | 1 | 0 | 0 | 1 | 0 | 0 | 1 | 0 | 0 | 1 | 0 | 0 |
| Unfavorable  outcomes | Win | none | 0 | 1 | 1 | 0 | 1 | 1 | 0 | 1 | 1 | 0 | 1 | 1 |
|  |  | small | 1 | 0 | 0 | 1 | 0 | 0 | 1 | 0 | 0 | 1 | 0 | 0 |
|  |  | large | 1 | 0 | 0 | 1 | 0 | 0 | 1 | 0 | 0 | 1 | 0 | 0 |
|  | Loss | none | 0 | 1 | 1 | 0 | 1 | 1 | 0 | 1 | 1 | 0 | 1 | 1 |
|  |  | small | 1 | 0 | 0 | 1 | 0 | 0 | 1 | 0 | 0 | 1 | 0 | 0 |
|  |  | large | 1 | 0 | 0 | 1 | 0 | 0 | 1 | 0 | 0 | 1 | 0 | 0 |

**Simple Models for Magnitude (specific, none + small vs. large)**

|  |  |  | Favorable Outcomes | | | | | | Unfavorable Outcomes | | | | | |
| --- | --- | --- | --- | --- | --- | --- | --- | --- | --- | --- | --- | --- | --- | --- |
|  |  |  | Win | | | Loss | | | Win | | | Loss | | |
|  |  |  | none | small | large | none | small | large | none | small | large | none | small | large |
| Favorable  Outcomes | Win | none | 0 | 0 | 1 | 0 | 0 | 1 | 0 | 0 | 1 | 0 | 0 | 1 |
|  |  | small | 0 | 0 | 1 | 0 | 0 | 1 | 0 | 0 | 1 | 0 | 0 | 1 |
|  |  | large | 1 | 1 | 0 | 1 | 1 | 0 | 1 | 1 | 0 | 1 | 1 | 0 |
|  | Loss | none | 0 | 0 | 1 | 0 | 0 | 1 | 0 | 0 | 1 | 0 | 0 | 1 |
|  |  | small | 0 | 0 | 1 | 0 | 0 | 1 | 0 | 0 | 1 | 0 | 0 | 1 |
|  |  | large | 1 | 1 | 0 | 1 | 1 | 0 | 1 | 1 | 0 | 1 | 1 | 0 |
| Unfavorable  outcomes | Win | none | 0 | 0 | 1 | 0 | 0 | 1 | 0 | 0 | 1 | 0 | 0 | 1 |
|  |  | small | 0 | 0 | 1 | 0 | 0 | 1 | 0 | 0 | 1 | 0 | 0 | 1 |
|  |  | large | 1 | 1 | 0 | 1 | 1 | 0 | 1 | 1 | 0 | 1 | 1 | 0 |
|  | Loss | none | 0 | 0 | 1 | 0 | 0 | 1 | 0 | 0 | 1 | 0 | 0 | 1 |
|  |  | small | 0 | 0 | 1 | 0 | 0 | 1 | 0 | 0 | 1 | 0 | 0 | 1 |
|  |  | large | 1 | 1 | 0 | 1 | 1 | 0 | 1 | 1 | 0 | 1 | 1 | 0 |

**Complex Models for Magnitude (overall)**

|  |  |  | Favorable Outcomes | | | | | | Unfavorable Outcomes | | | | | |
| --- | --- | --- | --- | --- | --- | --- | --- | --- | --- | --- | --- | --- | --- | --- |
|  |  |  | Win | | | Loss | | | Win | | | Loss | | |
|  |  |  | none | small | large | none | small | large | none | small | large | none | small | large |
| Favorable  Outcomes | Win | none | 0 | 0.05 | 0.5 | 0 | 0.05 | 0.5 | 0 | 0.05 | 0.5 | 0 | 0.05 | 0.5 |
|  |  | small | 0.05 | 0 | 0.45 | 0.05 | 0 | 0.45 | 0.05 | 0 | 0.45 | 0.05 | 0 | 0.45 |
|  |  | large | 0.5 | 0.45 | 0 | 0.5 | 0.45 | 0 | 0.5 | 0.45 | 0 | 0.5 | 0.45 | 0 |
|  | Loss | none | 0 | 0.05 | 0.5 | 0 | 0.05 | 0.5 | 0 | 0.05 | 0.5 | 0 | 0.05 | 0.5 |
|  |  | small | 0.05 | 0 | 0.45 | 0.05 | 0 | 0.45 | 0.05 | 0 | 0.45 | 0.05 | 0 | 0.45 |
|  |  | large | 0.5 | 0.45 | 0 | 0.5 | 0.45 | 0 | 0.5 | 0.45 | 0 | 0.5 | 0.45 | 0 |
| Unfavorable  outcomes | Win | none | 0 | 0.05 | 0.5 | 0 | 0.05 | 0.5 | 0 | 0.05 | 0.5 | 0 | 0.05 | 0.5 |
|  |  | small | 0.05 | 0 | 0.45 | 0.05 | 0 | 0.45 | 0.05 | 0 | 0.45 | 0.05 | 0 | 0.45 |
|  |  | large | 0.5 | 0.45 | 0 | 0.5 | 0.45 | 0 | 0.5 | 0.45 | 0 | 0.5 | 0.45 | 0 |
|  | Loss | none | 0 | 0.05 | 0.5 | 0 | 0.05 | 0.5 | 0 | 0.05 | 0.5 | 0 | 0.05 | 0.5 |
|  |  | small | 0.05 | 0 | 0.45 | 0.05 | 0 | 0.45 | 0.05 | 0 | 0.45 | 0.05 | 0 | 0.45 |
|  |  | large | 0.5 | 0.45 | 0 | 0.5 | 0.45 | 0 | 0.5 | 0.45 | 0 | 0.5 | 0.45 | 0 |

**Simple Models for Outcome (overall)**

|  |  |  | Favorable Outcomes | | | | | | Unfavorable Outcomes | | | | | |
| --- | --- | --- | --- | --- | --- | --- | --- | --- | --- | --- | --- | --- | --- | --- |
|  |  |  | Win | | | Loss | | | Win | | | Loss | | |
|  |  |  | none | small | large | none | small | large | none | small | large | none | small | large |
| Favorable  Outcomes | Win | none | 0 | 0 | 0 | 0 | 0 | 0 | 1 | 1 | 1 | 1 | 1 | 1 |
|  |  | small | 0 | 0 | 0 | 0 | 0 | 0 | 1 | 1 | 1 | 1 | 1 | 1 |
|  |  | large | 0 | 0 | 0 | 0 | 0 | 0 | 1 | 1 | 1 | 1 | 1 | 1 |
|  | Loss | none | 0 | 0 | 0 | 0 | 0 | 0 | 1 | 1 | 1 | 1 | 1 | 1 |
|  |  | small | 0 | 0 | 0 | 0 | 0 | 0 | 1 | 1 | 1 | 1 | 1 | 1 |
|  |  | large | 0 | 0 | 0 | 0 | 0 | 0 | 1 | 1 | 1 | 1 | 1 | 1 |
| Unfavorable  outcomes | Win | none | 1 | 1 | 1 | 1 | 1 | 1 | 0 | 0 | 0 | 0 | 0 | 0 |
|  |  | small | 1 | 1 | 1 | 1 | 1 | 1 | 0 | 0 | 0 | 0 | 0 | 0 |
|  |  | large | 1 | 1 | 1 | 1 | 1 | 1 | 0 | 0 | 0 | 0 | 0 | 0 |
|  | Loss | none | 1 | 1 | 1 | 1 | 1 | 1 | 0 | 0 | 0 | 0 | 0 | 0 |
|  |  | small | 1 | 1 | 1 | 1 | 1 | 1 | 0 | 0 | 0 | 0 | 0 | 0 |
|  |  | large | 1 | 1 | 1 | 1 | 1 | 1 | 0 | 0 | 0 | 0 | 0 | 0 |

**Simple Models for Outcome (specific, Favorable)**

|  |  |  | Favorable Outcomes | | | | | | Unfavorable Outcomes | | | | | |
| --- | --- | --- | --- | --- | --- | --- | --- | --- | --- | --- | --- | --- | --- | --- |
|  |  |  | Win | | | Loss | | | Win | | | Loss | | |
|  |  |  | none | small | large | none | small | large | none | small | large | none | small | large |
| Favorable  Outcomes | Win | none | 0 | 0 | 0 | 0 | 0 | 0 | 1 | 1 | 1 | 1 | 1 | 1 |
|  |  | small | 0 | 0 | 0 | 0 | 0 | 0 | 1 | 1 | 1 | 1 | 1 | 1 |
|  |  | large | 0 | 0 | 0 | 0 | 0 | 0 | 1 | 1 | 1 | 1 | 1 | 1 |
|  | Loss | none | 0 | 0 | 0 | 0 | 0 | 0 | 1 | 1 | 1 | 1 | 1 | 1 |
|  |  | small | 0 | 0 | 0 | 0 | 0 | 0 | 1 | 1 | 1 | 1 | 1 | 1 |
|  |  | large | 0 | 0 | 0 | 0 | 0 | 0 | 1 | 1 | 1 | 1 | 1 | 1 |
| Unfavorable  outcomes | Win | none | 1 | 1 | 1 | 1 | 1 | 1 | 1 | 1 | 1 | 1 | 1 | 1 |
|  |  | small | 1 | 1 | 1 | 1 | 1 | 1 | 1 | 1 | 1 | 1 | 1 | 1 |
|  |  | large | 1 | 1 | 1 | 1 | 1 | 1 | 1 | 1 | 1 | 1 | 1 | 1 |
|  | Loss | none | 1 | 1 | 1 | 1 | 1 | 1 | 1 | 1 | 1 | 1 | 1 | 1 |
|  |  | small | 1 | 1 | 1 | 1 | 1 | 1 | 1 | 1 | 1 | 1 | 1 | 1 |
|  |  | large | 1 | 1 | 1 | 1 | 1 | 1 | 1 | 1 | 1 | 1 | 1 | 1 |

**Simple Models for Outcome (specific, Unfavorable)**

|  |  |  | Favorable Outcomes | | | | | | Unfavorable Outcomes | | | | | |
| --- | --- | --- | --- | --- | --- | --- | --- | --- | --- | --- | --- | --- | --- | --- |
|  |  |  | Win | | | Loss | | | Win | | | Loss | | |
|  |  |  | none | small | large | none | small | large | none | small | large | none | small | large |
| Favorable  Outcomes | Win | none | 1 | 1 | 1 | 1 | 1 | 1 | 1 | 1 | 1 | 1 | 1 | 1 |
|  |  | small | 1 | 1 | 1 | 1 | 1 | 1 | 1 | 1 | 1 | 1 | 1 | 1 |
|  |  | large | 1 | 1 | 1 | 1 | 1 | 1 | 1 | 1 | 1 | 1 | 1 | 1 |
|  | Loss | none | 1 | 1 | 1 | 1 | 1 | 1 | 1 | 1 | 1 | 1 | 1 | 1 |
|  |  | small | 1 | 1 | 1 | 1 | 1 | 1 | 1 | 1 | 1 | 1 | 1 | 1 |
|  |  | large | 1 | 1 | 1 | 1 | 1 | 1 | 1 | 1 | 1 | 1 | 1 | 1 |
| Unfavorable  outcomes | Win | none | 1 | 1 | 1 | 1 | 1 | 1 | 0 | 0 | 0 | 0 | 0 | 0 |
|  |  | small | 1 | 1 | 1 | 1 | 1 | 1 | 0 | 0 | 0 | 0 | 0 | 0 |
|  |  | large | 1 | 1 | 1 | 1 | 1 | 1 | 0 | 0 | 0 | 0 | 0 | 0 |
|  | Loss | none | 1 | 1 | 1 | 1 | 1 | 1 | 0 | 0 | 0 | 0 | 0 | 0 |
|  |  | small | 1 | 1 | 1 | 1 | 1 | 1 | 0 | 0 | 0 | 0 | 0 | 0 |
|  |  | large | 1 | 1 | 1 | 1 | 1 | 1 | 0 | 0 | 0 | 0 | 0 | 0 |

**Complex Models for Outcome (overall)**

|  |  |  | Favorable Outcomes | | | | | | Unfavorable Outcomes | | | | | |
| --- | --- | --- | --- | --- | --- | --- | --- | --- | --- | --- | --- | --- | --- | --- |
|  |  |  | Win | | | Loss | | | Win | | | Loss | | |
|  |  |  | none | small | large | none | small | large | none | small | large | none | small | large |
| Favorable  Outcomes | Win | none | 0 | 0.05 | 0.5 | 0 | 0.05 | 0.5 | 0 | 0.05 | 0.5 | 0 | 0.05 | 0.5 |
|  |  | small | 0.05 | 0 | 0.45 | 0.05 | 0 | 0.45 | 0.05 | 0.1 | 0.55 | 0.05 | 0.1 | 0.55 |
|  |  | large | 0.5 | 0.45 | 0 | 0.5 | 0.45 | 0 | 0.5 | 0.55 | 1 | 0.5 | 0.55 | 1 |
|  | Loss | none | 0 | 0.05 | 0.5 | 0 | 0.05 | 0.5 | 0 | 0.05 | 0.5 | 0 | 0.05 | 0.5 |
|  |  | small | 0.05 | 0 | 0.45 | 0.05 | 0 | 0.45 | 0.05 | 0.1 | 0.55 | 0.05 | 0.1 | 0.55 |
|  |  | large | 0.5 | 0.45 | 0 | 0.5 | 0.45 | 0 | 0.5 | 0.55 | 1 | 0.5 | 0.55 | 1 |
| Unfavorable  outcomes | Win | none | 0 | 0.05 | 0.5 | 0 | 0.05 | 0.5 | 0 | 0.05 | 0.5 | 0 | 0.05 | 0.5 |
|  |  | small | 0.05 | 0.1 | 0.55 | 0.05 | 0.1 | 0.55 | 0.05 | 0 | 0.45 | 0.05 | 0 | 0.45 |
|  |  | large | 0.5 | 0.55 | 1 | 0.5 | 0.55 | 1 | 0.5 | 0.45 | 0 | 0.5 | 0.45 | 0 |
|  | Loss | none | 0 | 0.05 | 0.5 | 0 | 0.05 | 0.5 | 0 | 0.05 | 0.5 | 0 | 0.05 | 0.5 |
|  |  | small | 0.05 | 0.1 | 0.55 | 0.05 | 0.1 | 0.55 | 0.05 | 0 | 0.45 | 0.05 | 0 | 0.45 |
|  |  | large | 0.5 | 0.55 | 1 | 0.5 | 0.55 | 1 | 0.5 | 0.45 | 0 | 0.5 | 0.45 | 0 |

**Simple Models for Valence (overall)**

|  |  |  | Favorable Outcomes | | | | | | Unfavorable Outcomes | | | | | |
| --- | --- | --- | --- | --- | --- | --- | --- | --- | --- | --- | --- | --- | --- | --- |
|  |  |  | Win | | | Loss | | | Win | | | Loss | | |
|  |  |  | none | small | large | none | small | large | none | small | large | none | small | large |
| Favorable  Outcomes | Win | none | 0 | 0 | 0 | 1 | 1 | 1 | 0 | 0 | 0 | 1 | 1 | 1 |
|  |  | small | 0 | 0 | 0 | 1 | 1 | 1 | 0 | 0 | 0 | 1 | 1 | 1 |
|  |  | large | 0 | 0 | 0 | 1 | 1 | 1 | 0 | 0 | 0 | 1 | 1 | 1 |
|  | Loss | none | 1 | 1 | 1 | 0 | 0 | 0 | 1 | 1 | 1 | 0 | 0 | 0 |
|  |  | small | 1 | 1 | 1 | 0 | 0 | 0 | 1 | 1 | 1 | 0 | 0 | 0 |
|  |  | large | 1 | 1 | 1 | 0 | 0 | 0 | 1 | 1 | 1 | 0 | 0 | 0 |
| Unfavorable  outcomes | Win | none | 0 | 0 | 0 | 1 | 1 | 1 | 0 | 0 | 0 | 1 | 1 | 1 |
|  |  | small | 0 | 0 | 0 | 1 | 1 | 1 | 0 | 0 | 0 | 1 | 1 | 1 |
|  |  | large | 0 | 0 | 0 | 1 | 1 | 1 | 0 | 0 | 0 | 1 | 1 | 1 |
|  | Loss | none | 1 | 1 | 1 | 0 | 0 | 0 | 1 | 1 | 1 | 0 | 0 | 0 |
|  |  | small | 1 | 1 | 1 | 0 | 0 | 0 | 1 | 1 | 1 | 0 | 0 | 0 |
|  |  | large | 1 | 1 | 1 | 0 | 0 | 0 | 1 | 1 | 1 | 0 | 0 | 0 |

**Simple Models for Valence (specific, win)**

|  |  |  | Favorable Outcomes | | | | | | Unfavorable Outcomes | | | | | |
| --- | --- | --- | --- | --- | --- | --- | --- | --- | --- | --- | --- | --- | --- | --- |
|  |  |  | Win | | | Loss | | | Win | | | Loss | | |
|  |  |  | none | small | large | none | small | large | none | small | large | none | small | large |
| Favorable  Outcomes | Win | none | 0 | 0 | 0 | 1 | 1 | 1 | 0 | 0 | 0 | 1 | 1 | 1 |
|  |  | small | 0 | 0 | 0 | 1 | 1 | 1 | 0 | 0 | 0 | 1 | 1 | 1 |
|  |  | large | 0 | 0 | 0 | 1 | 1 | 1 | 0 | 0 | 0 | 1 | 1 | 1 |
|  | Loss | none | 1 | 1 | 1 | 0 | 1 | 1 | 1 | 1 | 1 | 1 | 1 | 1 |
|  |  | small | 1 | 1 | 1 | 1 | 0 | 1 | 1 | 1 | 1 | 1 | 1 | 1 |
|  |  | large | 1 | 1 | 1 | 1 | 1 | 0 | 1 | 1 | 1 | 1 | 1 | 1 |
| Unfavorable  outcomes | Win | none | 0 | 0 | 0 | 1 | 1 | 1 | 0 | 0 | 0 | 1 | 1 | 1 |
|  |  | small | 0 | 0 | 0 | 1 | 1 | 1 | 0 | 0 | 0 | 1 | 1 | 1 |
|  |  | large | 0 | 0 | 0 | 1 | 1 | 1 | 0 | 0 | 0 | 1 | 1 | 1 |
|  | Loss | none | 1 | 1 | 1 | 1 | 1 | 1 | 1 | 1 | 1 | 0 | 1 | 1 |
|  |  | small | 1 | 1 | 1 | 1 | 1 | 1 | 1 | 1 | 1 | 1 | 0 | 1 |
|  |  | large | 1 | 1 | 1 | 1 | 1 | 1 | 1 | 1 | 1 | 1 | 1 | 0 |

**Simple Models for Valence (specific, loss)**

|  |  |  | Favorable Outcomes | | | | | | Unfavorable Outcomes | | | | | |
| --- | --- | --- | --- | --- | --- | --- | --- | --- | --- | --- | --- | --- | --- | --- |
|  |  |  | Win | | | Loss | | | Win | | | Loss | | |
|  |  |  | none | small | large | none | small | large | none | small | large | none | small | large |
| Favorable  Outcomes | Win | none | 0 | 1 | 1 | 1 | 1 | 1 | 1 | 1 | 1 | 1 | 1 | 1 |
|  |  | small | 1 | 0 | 1 | 1 | 1 | 1 | 1 | 1 | 1 | 1 | 1 | 1 |
|  |  | large | 1 | 1 | 0 | 1 | 1 | 1 | 1 | 1 | 1 | 1 | 1 | 1 |
|  | Loss | none | 1 | 1 | 1 | 0 | 0 | 0 | 1 | 1 | 1 | 0 | 0 | 0 |
|  |  | small | 1 | 1 | 1 | 0 | 0 | 0 | 1 | 1 | 1 | 0 | 0 | 0 |
|  |  | large | 1 | 1 | 1 | 0 | 0 | 0 | 1 | 1 | 1 | 0 | 0 | 0 |
| Unfavorable  outcomes | Win | none | 1 | 1 | 1 | 1 | 1 | 1 | 0 | 1 | 1 | 1 | 1 | 1 |
|  |  | small | 1 | 1 | 1 | 1 | 1 | 1 | 1 | 0 | 1 | 1 | 1 | 1 |
|  |  | large | 1 | 1 | 1 | 1 | 1 | 1 | 1 | 1 | 0 | 1 | 1 | 1 |
|  | Loss | none | 1 | 1 | 1 | 0 | 0 | 0 | 1 | 1 | 1 | 0 | 0 | 0 |
|  |  | small | 1 | 1 | 1 | 0 | 0 | 0 | 1 | 1 | 1 | 0 | 0 | 0 |
|  |  | large | 1 | 1 | 1 | 0 | 0 | 0 | 1 | 1 | 1 | 0 | 0 | 0 |

**Complex Models for Valence (overall)**

|  |  |  | Favorable Outcomes | | | | | | Unfavorable Outcomes | | | | | |
| --- | --- | --- | --- | --- | --- | --- | --- | --- | --- | --- | --- | --- | --- | --- |
|  |  |  | Win | | | Loss | | | Win | | | Loss | | |
|  |  |  | none | small | large | none | small | large | none | small | large | none | small | large |
| Favorable  Outcomes | Win | none | 0 | 0.05 | 0.5 | 0 | 0.05 | 0.5 | 0 | 0.05 | 0.5 | 0 | 0.05 | 0.5 |
|  |  | small | 0.05 | 0 | 0.45 | 0.05 | 0.1 | 0.55 | 0.05 | 0 | 0.45 | 0.05 | 0.1 | 0.55 |
|  |  | large | 0.5 | 0.45 | 0 | 0.5 | 0.55 | 1 | 0.5 | 0.45 | 0 | 0.5 | 0.55 | 1 |
|  | Loss | none | 0 | 0.05 | 0.5 | 0 | 0.05 | 0.5 | 0 | 0.05 | 0.5 | 0 | 0.05 | 0.5 |
|  |  | small | 0.05 | 0.1 | 0.55 | 0.05 | 0 | 0.45 | 0.05 | 0.1 | 0.55 | 0.05 | 0 | 0.45 |
|  |  | large | 0.5 | 0.55 | 1 | 0.5 | 0.45 | 0 | 0.5 | 0.55 | 1 | 0.5 | 0.45 | 0 |
| Unfavorable  outcomes | Win | none | 0 | 0.05 | 0.5 | 0 | 0.05 | 0.5 | 0 | 0.05 | 0.5 | 0 | 0.05 | 0.5 |
|  |  | small | 0.05 | 0 | 0.45 | 0.05 | 0.1 | 0.55 | 0.05 | 0 | 0.45 | 0.05 | 0.1 | 0.55 |
|  |  | large | 0.5 | 0.45 | 0 | 0.5 | 0.55 | 1 | 0.5 | 0.45 | 0 | 0.5 | 0.55 | 1 |
|  | Loss | none | 0 | 0.05 | 0.5 | 0 | 0.05 | 0.5 | 0 | 0.05 | 0.5 | 0 | 0.05 | 0.5 |
|  |  | small | 0.05 | 0.1 | 0.55 | 0.05 | 0 | 0.45 | 0.05 | 0.1 | 0.55 | 0.05 | 0 | 0.45 |
|  |  | large | 0.5 | 0.55 | 1 | 0.5 | 0.45 | 0 | 0.5 | 0.55 | 1 | 0.5 | 0.45 | 0 |
